# Evaluation of deep learning‐based automated radiotherapy planning for early‐stage lung cancer using SBRT‐VMAT: A comparison with manual planning

**DOI:** 10.1002/acm2.70291

**Published:** 2025-10-14

**Authors:** Hikaru Nemoto, Masahide Saito, Noriyuki Kadoya, Takafumi Komiyama, Ryota Tozuka, Hiroshi Onishi, Keiichi Jingu

**Affiliations:** ^1^ Department of Radiation Oncology Tohoku University Graduate School of Medicine Sendai Miyagi Japan; ^2^ Department of Radiology University of Yamanashi Chuo‐city Yamanashi Japan

**Keywords:** deep learning, dose prediction, lung cancer, stereotactic body radiation therapy, volumetric modulated arc therapy

## Abstract

**Purpose:**

This study evaluates the feasibility of deep learning (DL)‐based automated SBRT‐VMAT planning for lung cancer.

**Methods:**

We developed a DL‐based dose distribution prediction model (installed in the RatoGuide prototype, AiRato. Inc.), which was trained using manually optimized and clinically approved plans for 124 cases of early‐stage lung cancer (62 central and 62 peripheral cases). The test data consisted of eight treatment plans for each case. Automated plans for the test data were created as follows: (i) predicting dose distributions from CT images with target and organs‐at‐risk contours using RatoGuide, (ii) converting the predicted dose distributions into ring‐shaped dose structures and importing them into RayStation software, and (iii) generating deliverable dose distributions automatically to reproduce the predicted dose distributions using a scripting application within the treatment planning system. We measured the time required to create the plans and compared the DVH metrics of the automated and manual plans. Two expert radiation oncologists evaluated the automated and manual plans based on following aspects: (a) clinical acceptability and (b) preference for automated or manual plans in clinical practice.

**Results:**

No significant differences were observed in any DVH metric between the automated and manual plans in each case. Two radiation oncologists reviewed all automated plans and deemed them clinically acceptable. The number of cases evaluated as the preferred automated plan by radiation oncologists was higher for central than peripheral cases.

**Conclusions:**

These results indicate the feasibility of automated treatment planning using a prediction model based on DL, suggesting that DL techniques can efficiently generate clinically acceptable SBRT‐VMAT treatment plans for lung cancer.

## 1 INTRODUCTION

Intensity modulated radiation therapy (IMRT) and volumetric‐modulated arc therapy (VMAT) can improve dose coverage to the planning target volume (PTV) and dose reduction to organs at risk (OARs).^1^, ^2^, ^3^ These highly conformal radiation therapies (RTs) are also used in stereotactic body radiation therapy (SBRT) and can deliver dose distributions with sharper dose gradients than conventional treatments.^4^, ^5^ IMRT/VMAT treatment plans are performed with complex multileaf collimator (MLC) positions and irradiation using dynamic dose rates. These plans also require iterative dose and optimization calculations to adjust multiple parameters, including the MLC positions. This iterative optimization‐based inverse treatment planning is time‐consuming and or labor‐intensive and requires hours or even days to complete. Specifically, the planner sets the initial treatment planning objectives for various structures and numerically defines the optimization objectives. The quality of treatment plans varies depending on the technology and knowledge of the planner for this optimization process.^6^, ^7^, ^8^ Nelms et al. evaluated IMRT treatment plans using a Plan Quality Metric and reported that plan quality varied regardless of the background factors of the planner.^9^ The quality of treatment plans can be improved to some extent by taking more time for planning; however, in actual clinical practice, the balance between the time spent planning and the quality of the plan should be considered. Therefore, the issue is how to guarantee consistency in the quality of treatment plans among institutions.

In recent years, several studies have been conducted on the development of artificial intelligence (AI)‐based automated RT planning systems to improve the quality of treatment planning and increase the efficiency of planning procedures. As automated RT planning systems continue to develop, the quality of the plans is expected to become less dependent on the planner, and improvements in the efficiency and consistency of the plans are expected. Dose distribution prediction using deep learning (DL) has been extensively studied, and the development of prediction models that can generate predicted dose distributions based on the input contours of targets such as PTVs and OARs has been reported.^10^, ^11^, ^12^ Koike et al. developed three‐dimensional (3D) voxel‐level dose distribution prediction models for prostate cancer and reported that the prediction error for the target was within 1% and that the prediction error for OARs was within 5%.^12^ The results of these dose prediction studies indicate the potential for improving the efficiency of treatment planning processes and the quality of treatment plans. Additionally, several methods for creating deliverable plans from predicted dose distributions using a radiation therapy planning system (RTPS) have been reported.^13^, ^14^, ^15^, ^16^, ^17^, ^18^, ^19^ Shen et al. developed a method for creating deliverable dose distributions optimized using finite elements based on the dose distribution predicted using DL for the treatment of prostate cancer.^13^ They integrated this approach with an open‐source RTPS to create deliverable plans. Church et al. optimized and created deliverable plans by importing an objective function derived from the predicted dose distribution for prostate cancer into a commercially available RTPS (RayStation).^14^ Kadoya et al. evaluated the feasibility of automated treatment planning using DL‐based dose distribution prediction software for prostate cancer,^17^ while Saito et al. evaluated the same approach for head and neck cancer.^18^ Wang et al. investigated the potential of automated treatment planning using DL for stage 1–4 lung cancer, and Bai et al. reported automated treatment planning methods using machine learning for early‐stage lung cancer.^15^, ^19^ However, the development of DL models for automated treatment planning and methods to create SBRT plans using VMAT for early‐stage lung cancer have not been thoroughly evaluated. Particularly, methods for automatically creating deliverable plans using commercial RTPS based on predicted dose distributions have not yet been sufficiently evaluated. This study aimed to evaluate the feasibility of DL‐based automated SBRT‐VMAT planning for lung cancer to improve the efficiency and quality of planning. This study was performed using novel software that can predict dose distribution.

## 2 MATERIALS AND METHODS

### 2.1 Ethics approval

This study was approved by the institutional review board of the University of Yamanashi. The receipt number was CS0010.

### 2.2 Patient characteristics and Treatment planning

This study included 124 patients with early‐stage lung cancer (62 peripheral and 62 central) who were retrospectively treated with SBRT using three‐dimensional conformal radiation therapy (3D‐CRT) or VMAT at our hospital. The treatment machine was Elekta Synergy with an Agility gantry head (Elekta AB; Stockholm, Sweden). The average gross tumor diameters of peripheral and central cases were 20.6 (7.0–52.6) mm and 22.1 (4.0–57.4) mm, respectively. Prescription doses and fractionation were 55 Gy / 4 fr for peripheral cases and 50 Gy / 8 fr for central cases, both prescribed to PTV D95%. The dose constraints for the PTV were D95%≥ the prescription dose, and the maximum dose (D2%) was intentionally increased to approximately 125%–130% of the prescription dose. The dose constraints for the planned OAR volumes are listed in Table 1. For peripheral cases, dose constraints were based on the Japan Clinical Oncology Group (JCOG) study 1408 protocol,^20^ For central cases, the dose constraints were based on the Japanese Radiation Oncology Study Group (JROSG) study 10–1 protocol.^21^ Clinically approved treatment plans for each case by expert radiation oncologists were manually optimized using RayStation ver. 10A (RaySearch Laboratories, Stockholm, Sweden) and were used for treatment. The dose calculation algorithm used was collapsed cone convolution version 5.3, and the dose calculation grid size was 2 mm. Each plan was created using a single partial‐arc VMAT. Two experienced medical physicists performed treatment planning.

**TABLE 1:** Dose constraints of organ at risk (OAR) for peripheral cases based on Japan Clinical Oncology Group (JCOG) 1408 protocol and for central cases based on Japanese Radiation Oncology Study Group (JROSG) 10‐1 protocol.

	Peripheral case (JCOG1408)			Central case (JROSG10‐1)		
PRV (planning organ at risk volume)	Objectives		Constraints	Objectives		Constraints
Lungs‐GTV or Lungs‐ITV	V40 Gy	≤	100 cc	V20 Gy	<	20%
	Dmean	≤	18 Gy			
	V15 Gy	≤	25 %			
	V20 Gy	≤	20 %			
Spinal cord +3 mm	Dmax	≤	25 Gy	Dmax	<	33.5 Gy
Esophagus +5 mm	D1cc	≤	40 Gy	V40 Gy	<	5 cc
Pulmonary artery +5 mm				V54.5 Gy	<	1 cc
	D10cc	≤	35 Gy	V47.5 Gy	<	10 cc
SVC, Pulmonary vein				V48 Gy	<	1 cc
Aorta				V58 Gy	<	10 cc
Heart +3 mm	D15cc	≤	30 Gy	V40 Gy	<	15 cc
Stomach +5 mm	D10cc	≤	36 Gy			
Bowel +5 mm	D100cc	≤	30 Gy			
Trachea +5 mm	D10cc	≤	40 Gy	V54.5 Gy	<	10 cc
Main Bronchus +5 mm	D10cc	≤	40 Gy	V54.5 Gy	<	10 cc
Brachial plexus +3 mm	D3cc	≤	25 Gy	Dmax	<	40 Gy
Skin				Dmax	<	40 Gy
Other organs +3 mm	D1cc	≤	48 Gy	V65.5 Gy	<	1 cc
	D10cc	≤	40 Gy	V54.5 Gy	<	10 cc

### 2.3 Automated creation of deliverable VMAT plans

**FIGURE 1:**
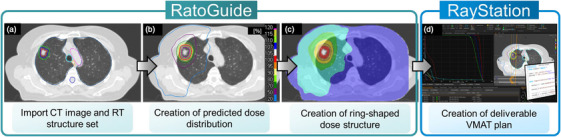
The outline of automated planning procedure using RatoGuide and RayStation. (a) DICOM data of planning CT and RT structure set were imported to RatoGuide software. (b) Dose distribution was predicted using RatoGuide. (c) RatoGuide automatically generated dose structures for optimization based on predicted dose. CT images and RT structure set were exported with dose structures to RayStation. (d) Automated treatment planning on RayStation using scripts. The calculated dose distributions were evaluated. CT, computed tomography; RT, radiation therapy.

An outline of the automated planning procedure is shown in Figure 1. The RatoGuide prototype software (AiRato. Inc., Sendai, Japan) was designed to predict dose distributions based on the input planning computed tomography (CT) images, RT structures of targets and normal organs, and the prescribed dose. The DL model installed in the RatoGuide prototype was trained using CT images, RT structures, and the dose distribution of clinically approved treatment plans for central and peripheral types in 54 cases. This DL model can predict dose distributions based on CT images and structure sets. It can also generate isodose line‐based structures for use in the optimization process to create deliverable treatment plans. The test data comprised eight treatment plans for central and peripheral lung cancer. These plans were manually optimized and used previously for treatment.

The automated plans for each test case were created using the following procedure. First, the CT and RT structure set data were imported into the RatoGuide prototype software. Second, the dose distribution (PredDose) was predicted based on the target, OAR contours, and CT images using the pretrained DL‐based model. However, the prediction is limited to the DICOM RT dose and does not constitute a deliverable treatment plan at this stage. This model was developed using UNet‐based 3D dose prediction, as proposed by Nguyen et al.^22^ Third, the PredDoses were converted into contours at 20% dose increments, and ring‐shaped dose structures for optimization were automatically created. The ring‐shaped dose structure used in this study was derived from PredDose by subtraction. Specifically, higher‐dose isodose volumes were subtracted from lower‐dose volumes, with each subtraction occurring in 20% increments. As shown in Table 2, “Dose Structure_20.0_40.0%” denotes a ring‐shaped structure that is generated as follows: isodose structures corresponding to 20% and 40% of the prescription dose are generated; the 40% dose structure is then subtracted from the 20% one. Finally, the CT and RT structure set data, including the target, OAR, and automatically created dose structures, were imported into RayStation. Subsequently, deliverable treatment plans that reproduced the PredDose were automatically generated in RayStation using custom‐developed scripts. The entire treatment planning process was automated using the RayStation Python application programming interface. This included the following actions: creating a new plan; selecting the treatment machine and photon energy; defining the prescription dose and type; adding arc beams with isocenter placement, gantry, and collimator angle settings; setting optimization objectives and their corresponding weights; performing optimization and normalization; and executing the final dose calculation. An integrated script that automatically executed the workflow described above was used to generate automated treatment plans for each test case. The script‐based optimization was performed in two sequential steps to minimize the difference between the optimized dose and the final dose calculation results. First, the fluence map optimization was performed for 70 iterations. An additional 30 iterations of optimization converting the fluence map into segments were then performed, followed by a final dose calculation. The second step consisted of additional 30 optimization iterations and a final dose calculation, considering the results of the first step. Finally, the automated plan was normalized to the prescribed dose. In the optimization process of the automated plans, only the ring‐shaped dose structures and the target structure were used as the objectives. Table 2 lists the specific optimization parameters and objectives for each of the ring‐shaped dose structures and target structures used to convert PredDose into deliverable plans. Moreover, the function values displayed in RayStation for each objective were recorded at the end of the optimization process. This value provides a quantitative measure of the degree to which the current treatment plan satisfies the specified optimization objectives. Lower values indicate greater conformity to the planned goals.

**TABLE 2:** Objectives and parameter settings used in script‐based optimization for both peripheral and central case.

Peripheral case			
ROI	Objectives	Value (Peripheral case)	Weight
PTV	Min DVH	5500 cGy to 95 % volume	100
PTV	Max dose	6875 cGy	100
Dose structure_100.0_120.0%	Min DVH	6050 cGy to 50% volume	1
Dose structure_80.0_100.0%	Max dose	5500 cGy	1
Dose structure_60.0_80.0%	Max dose	4400 cGy	1
Dose structure_40.0_60.0%	Max dose	3300 cGy	1
Dose structure_20.0_40.0%	Max dose	2200 cGy	1
Dose structure_0.0_20.0%	Max dose	1100 cGy	1

Central case			
ROI	Objectives	Value (Central case)	Weight
PTV	Min DVH	5000 cGy to 95% volume	100
PTV	Max dose	6000 cGy	100
Dose structure_100.0_120.0%	Min DVH	5500 cGy to 50% volume	1
Dose structure_80.0_100.0%	Max dose	5000 cGy	1
Dose structure_60.0_80.0%	Max dose	4000 cGy	1
Dose structure_40.0_60.0%	Max dose	3000 cGy	1
Dose structure_20.0_40.0%	Max dose	2000 cGy	1
Dose structure_0.0_20.0%	Max dose	1000 cGy	1

*Note*: The notation “Dose structure_A_B%” in the table indicates that they are ring‐shaped dose structures ranging from A% Rx dose to B% Rx dose based on predicted dose distribution.

Abbreviations: DVH, dose volume histogram; PTV, planning target volume.

### 2.4 Objective evaluation of dose distributions

We measured and compared DVH metrics between the automated and manual plans. First, the following DVH metrics and parameter were evaluated: D2% and D98% of the PTV, average dose, V20Gy and V5Gy of lungs‐GTV, maximum dose of spinal cord, maximum dose of the skin, monitor units (MU), the Paddick conformity index (CI),^23^ and the RTOG CI and ratio of 50% prescription isodose volume to PTV (R50).^24^, ^25^, ^26^ We performed statistical analysis using the Wilcoxon signed‐rank test for all DVH metrics between the automated and manual plans. Second, the time required to create each automated plan was measured. The time for automated planning was defined as the time from the start of the workflow in RatoGuide to the completion of treatment planning in RayStation. Third, we used the Dice similarity coefficients (DSC) to evaluate the agreement in dose distributions between automated plans and PredDose. Specifically, to assess the reproducibility of the PredDose on the RTPS using the automated plan, we created dose structures at 20% dose intervals for each distribution, and the DSC was calculated.

### 2.5 Subjective evaluation of automated plans

Two expert radiation oncologists evaluated the automated and manual plans based on visual inspection and DVH metrics for the following aspects: (a) clinical acceptability and (b) preference for automated or manual plans in clinical practice. Evaluators were blinded as to whether the plan was created automatically or manually. Specifically, two expert radiation oncologists first assessed the clinical acceptability of the treatment plans based on whether all dose constraints for the targets and organs at risk were satisfied in accordance with the JCOG1408 and JROSG10‐1 protocols. They then independently performed a qualitative evaluation of each plan, considering factors not defined in the protocols, such as DVH metrics, overall shape of the dose distribution, and dose convergence. This evaluation method was designed to assess the robustness of the automated plans in the context of the variability in clinical judgment among evaluators.^27^, ^28^

## 3 RESULTS

### 3.1 Dose distribution and DVH

**FIGURE 2:**
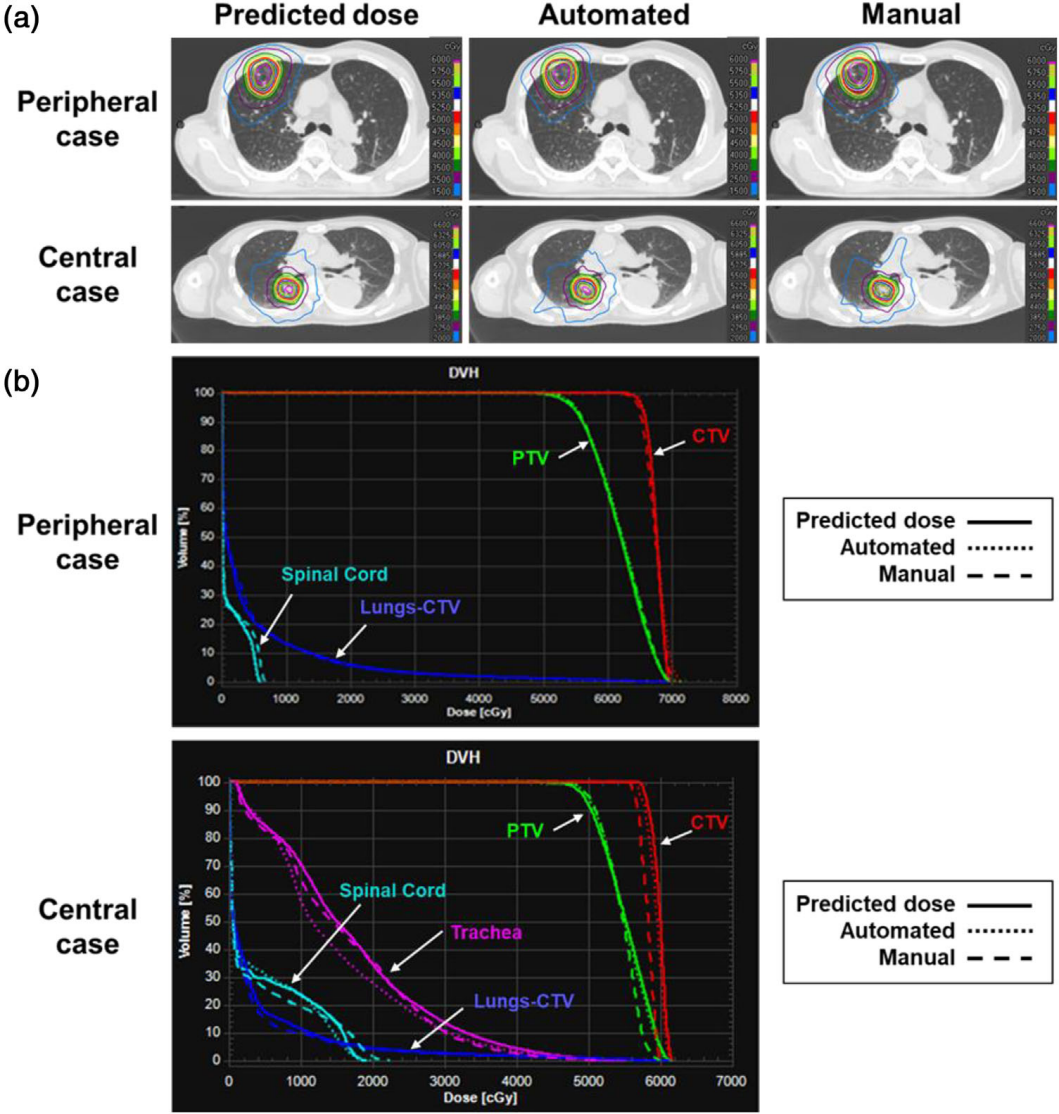
Dose distribution and DVH for representative cases of both central and peripheral cancers. (a) The dose distribution of the predicted, automated, and manual plan for each central and peripheral case. (b) The DVHs of the predicted, automated, and manual plan for each central and peripheral case. DVH, dose volume histogram.

Figure 2 shows the automatically and manually generated dose distributions for test case 3 for peripheral lung cancer and test case 1 for central lung cancer. Each dose distribution image shows the axial plane of the isocenter. The images on the left in Figure 2 show the dose distributions predicted using RatoGuide. However, PredDose could not be delivered because no MLC position information was available. The DVH for each plan is shown at the bottom of the figure. The dose distribution and DVH for peripheral cases generally agreed with those in the manual plan. However, for the dose distribution of the central cases, good agreement was found in the high‐dose region around the target; however, slight differences were observed in the mid‐ to low‐dose regions. Table S1 shows the function values displayed in RayStation at the end of the optimization for each objective used to generate the script‐based automated plan. The maximum dose functions for the PTV and the two types of dose structures for the mid to low‐dose regions exhibited the highest function values.

### 3.2 Objective evaluation of dose distributions

**FIGURE 3:**
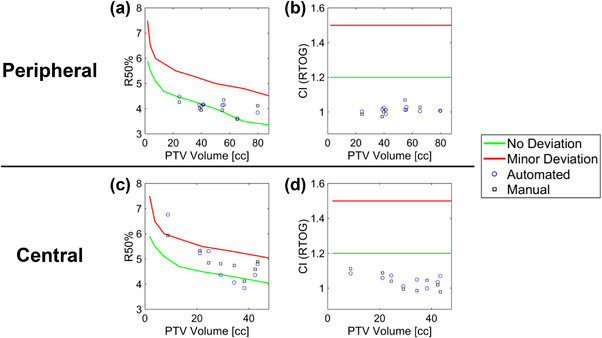
Plots show the RTOG R50 (a, c) and CI (b, d) for peripheral (upper) and central (lower) cases, comparing automated (blue circles) and manual (black squares) plans. The solid green and red lines indicate the RTOG criteria for no deviation and minor deviation, respectively. All CI values met the minor deviation criteria. All R50 values for manual plans met the minor deviation criteria, while all automated plans met the minor deviation criteria except for one peripheral case. CI, conformity index; RTOG, Radiation Therapy Oncology Group.

**FIGURE 4:**
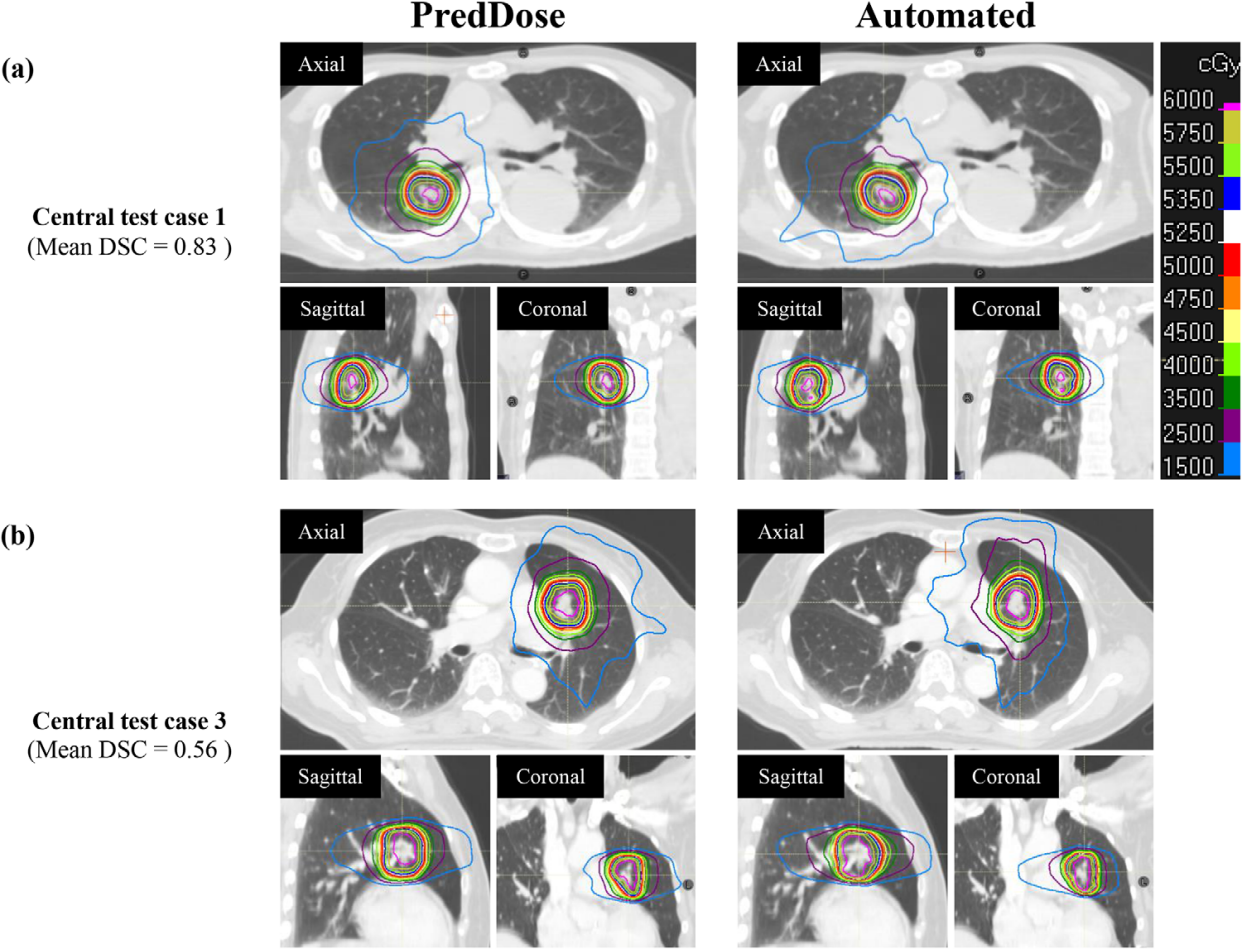
Dose distributions for typical cases with good (a) and poor (b) average DSC across the evaluated 20%–100% dose structures between the predicted dose distribution (PredDose) and the automatically created deliverable dose distribution (Automated). DSC, dice coefficients; PredDose, predicted dose distribution.

Table 3 shows DVH metrics and creation time for the automated and manually created plans. For each case, no significant differences were observed between the DVH metrics of the automated and manual plans. The average MU were lower in the automated plans than the manual plans, with a significantly lower MU observed in the central cases. No significant differences were observed between the automated and manual plans in the two types of CI and R50. Figure 3 shows scatter plots of the RTOG R50 and CI values for the automated and manual plans. All automated and manual plans met the defined criterion of ≤1.2 for the RTOG CI^26^ for each case. The compliance rate for the RTOG R50 criteria was 100% for peripheral cases and 88% for central cases, including minor deviations. Table 4 presents the mean and standard deviation of the DSC between the PredDose and automated dose distributions per 20% dose structure. In central and peripheral cases, the highest DSC values were observed in the high‐dose regions (80%–100% dose structures), while slightly lower DSC values were observed in the mid‐to low‐dose regions (20%–80% dose structures). Additionally, the DSC values were generally slightly lower in central cases than in peripheral cases. Figure 4 shows test cases with good and poor agreement between the PredDose structures and the dose structures of the automated plans.

**TABLE 3:** Mean ± standard deviation of the dose volume histogram (DVH) metrics, Paddick conformity index (CI_Paddick_), RTOG conformity index (CI_RTOG_), ratio of 50% prescription isodose volume to PTV (R50), and creation time for manual and automated plans.

		Peripheral			Central		
		Manual	Automated	*p‐*value	Manual	Automated	*p‐*value
PTV	D98% (Gy)	53.26 ± 0.36	53.47 ± 0.38	0.46	48.28 ± 0.51	48.12 ± 0.41	0.55
	D2% (Gy)	70.27 ± 0.91	68.55 ± 17.53	0.08	61.17 ± 2.16	61.04 ± 0.50	1.00
Lungs‐GTV	V20Gy (%)	3.83 ± 1.51	3.85 ± 1.43	0.95	5.79 ± 1.65	5.79 ± 1.55	0.97
	V5Gy (%)	13.32 ± 3.93	16.24 ± 6.45	0.20	18.92 ± 5.74	23.87 ± 8.18	0.25
	Dmean (Gy)	3.09 ± 0.91	3.24 ± 0.98	0.46	4.15 ± 0.87	4.55 ± 1.01	0.20
SpinalCord	Dmax (Gy)	8.29 ± 3.10	10.64 ± 4.81	0.08	15.37 ± 6.54	17.05 ± 7.85	0.25
Skin	Dmax (Gy)	23.55 ± 7.70	25.41 ± 7.14	0.11	19.69 ± 4.45	20.49 ± 3.80	0.46
CI_Paddick_		0.87 ± 0.04	0.87 ± 0.02	0.64	0.90 ± 0.02	0.90 ± 0.04	0.74
CI_RTOG_		1.05 ± 0.05	1.05 ± 0.03	0.54	1.01 ± 0.03	1.01 ± 0.01	1.00
R50_RTOG_		4.95 ± 0.63	4.84 ± 0.94	0.64	4.05 ± 0.24	4.08 ± 0.26	0.64
Creation time (min)		–	4.78 ± 1.17	–	–	4.57 ± 0.80	–
MU		2458.13	2447.04	0.95	1423.33	1301.30	0.02*

*Note*: The average creation time and average monitor unit (MU) for each plan were shown at the bottom of the table.

Abbreviations: PTV, planning target volume; RTOG, Radiation Therapy Oncology Group.

**TABLE 4:** Mean ± standard deviation (SD) of dice coefficients (DSC) between the predicted dose distribution (PredDose) and the automatically created deliverable plan using ring‐shaped structures (Automated) per 20% dose structure.

Dose structure	Peripheral	Central
	PredDose versus Automated	PredDose versus Automated
20%–40%	0.74 ± 0.086	0.74 ± 0.133
40%–60%	0.75 ± 0.056	0.72 ± 0.107
60%–80%	0.75 ± 0.052	0.73 ± 0.076
80%–100%	0.80 ± 0.042	0.77 ± 0.064
Overall (20%–100%)	0.74 ± 0.096	0.76 ± 0.062

### 3.3 Subjective evaluation of automated plans

Table 5 shows the results of subjective evaluations by the two radiation oncologists. The percentages of cases in which the two radiation oncologists preferred automated plans over manual plans were 62.5% and 37.5%, respectively, for peripheral cases, and 68.75% and 37.5%, respectively, for central cases. The number of cases evaluated as preferred automated plans over manual plans by radiation oncologists was higher for central than for peripheral cases. Regarding whether these plans could be used in clinical practice, all automated treatment plans were evaluated as acceptable.

**TABLE 5:** Summary of subjective evaluation by radiation oncologists (RO).

	Which is preferable, a manual plan or an automated plan?			
	Peripheral cases		Central cases	
	RO1	RO2	RO1	RO2
Test case 1	Automated	Manual	Automated	Automated
Test case 2	Automated	Automated	Parity*	Manual
Test case 3	Automated	Manual	Automated	Automated
Test case 4	Manual	Manual	Automated	Manual
Test case 5	Automated	Automated	Automated	Manual
Test case 6	Manual	Manual	Automated	Automated
Test case 7	Automated	Automated	Manual	Manual
Test case 8	Manual	Manual	Manual	Manual
The percentage of cases chosen automated plan	62.5%	37.5%	68.75%	37.5%

## 4 DISCUSSION

Various studies have been performed to predict dose distributions using CT images and RT structures, apply the results to treatment planning, improve the accuracy of prediction models, and standardize the methods that have been performed.^16^, ^17^, ^19^, ^29^ Wang et al. evaluated automated treatment planning for lung cancer in stages 1–4.^15^ Regarding the quality of automated treatment planning, they compared the automated plans and manually created plans and reported that although target coverage was not significantly different, significant differences were observed in V5Gy of the lungs (1.8%, *p *= 0.02) and maximum dose of the spinal cord (0.7 Gy, *p *= 0.04). However, our study focused on SBRT for the treatment of lung cancer. No significant differences were observed in the DVH metrics for the targets or OAR. Similarly, no significant differences were observed in any OAR dose metric between the manual and automated treatment plans in previous study.^30^

As shown in Table 4, DSC was used to quantitatively evaluate the similarity between the PredDose and automated dose distributions in this study. The DSC, a metric commonly used to assess spatial overlap, was calculated for various isodose volumes (e.g., 40%–60% and 20%–40% for both the PredDose and the automated dose distributions) to evaluate the reproducibility of the PredDose for automated plans. The DSC values were slightly lower for central‐type tumors, probably indicating the reduced reproducibility of PredDose in anatomically complex regions. Despite a tendency for a modest reduction in agreement between PredDose and automatically planned doses in the low‐ to mid‐dose regions compared with that in the high dose regions, all OAR constraints defined by the JCOG1408 and JROSG10‐1 protocols^20^, ^21^ were met in every case. However, recognizing that such discrepancies in dose distribution may have clinical implications, further studies are required to clarify their potential long‐term effects. Moreover, the function values for the maximum dose objectives applied to the dose structures were relatively high in the mid‐to‐low dose regions (0%–60%). This result indicates that the optimization in these regions may not have fully converged during the creation of automated plans compared with other dose levels. This finding is consistent with the slightly lower DSC values observed in these regions. Collectively, these results suggest that the DL model may have overestimated the steepness of dose gradients in these regions. Alternatively, these results may reflect the inherent limitations of planning methods using ring‐shaped dose structures.

Knowledge‐based planning (KBP) systems such as RapidPlan (RP) (Varian Medical System) are currently in use, and studies have reported that KBP‐generated plans are equivalent in quality to clinical plans.^31^, ^32^, ^33^ Visak et al. evaluated the performance of KBP for central and peripheral lung cancer and showed that plans with equivalent quality to clinical plans could be generated for PTV D99%, CI, lung V20, V5Gy, and mean dose. However, the MUs for the KBP plan were significantly 24% higher than for the manual plan.^31^, ^32^ Our findings are consistent with those of previous studies in that no significant differences were observed between the automated and manual plans across all DVH metrics. Further studies are needed to directly compare DVH metrics between the KBP plans and automated plans. However, the observation of a significant 8.5% decrease in MU in the automated plans contrasts with trends reported in previous studies.^31^, ^32^, ^34^ A possible reason for the disagreement with the results of the MU in this study is due to the difference in the optimization method. Optimization using RatoGuide employs a ring‐shaped dose structure, which may make it easier for the objectives to converge. Specifically, using ring‐shaped dose structures derived from the isodose lines of PredDose, which are generated by a DL‐based prediction model, as optimization goals help clarify the direction of optimization in the RTPS. This approach can potentially reduce the number of iterations required. Additionally, defining specific dose constraints for each ring‐shaped dose structure probably narrows the search space, suppressing unnecessary exploration and enabling faster convergence than conventional optimization methods. In this study, optimizing the automated plan required only two sets of 30 iterations to create plans with no significant differences in DVH metrics compared with plans that were manually optimized with a larger number of iterations. Therefore, the automated planning approach employed in this study may have contributed to the reduction in the MU by suppressing excessive modulation complexity. Although a decrease in the MU of the automated plan may imply better delivery efficiency, it could also be associated with reduced modulation complexity, resulting in a lower plan quality. This is crucial in central cases, where steep dose gradients are needed to spare adjacent organs at risk. Although our analysis revealed no significant differences in DVH metrics for targets or OARs, the DSC values in Table 4 tended to be lower for central cases than for peripheral cases. This suggests a potential decline in the PredDose reproducibility. These results emphasize the importance of investigating the relationship among MU reduction, modulation complexity, and dose delivery accuracy, particularly in anatomically complex cases. Future research should also consider evaluating additional metrics, such as modulation complexity scores, to evaluate the plan quality more comprehensively. Regarding the increase in MU of KBP, treatment planning using machine learning tends to be complex.^35^ Kubo et al. used RP (Varian Medical System, a KBP system that uses machine learning) to evaluate the modulation complexity score for VMAT (MCSv) between manual plans and automated plans using RP. They showed that plans using RP have the potential to create plans that are more complex than manual plans.^35^

The time required to create the treatment plan in this study was 4.57 and 4.78 min on average for each central and peripheral case, respectively. In previous studies, it was 30 min, 32.6 min and 40–60 min on average.^15^, ^19^, ^31^ In our clinical practice, the time required to complete a VMAT SBRT plan of 50 Gy /8 fr or 55 Gy / 4 fr for early‐stage lung cancer is approximately 30–60 min. Therefore, the automatic planning method in this study demonstrated the possibility of creating a treatment plan for early‐stage lung cancer more quickly. This will enable planners to save a significant amount of time.^36^ This also indicates the possibility of reducing the number of days required to prepare for VMAT using automated planning for lung cancer.

To the best of our knowledge, no study has focused on automatically creating deliverable VMAT treatment plans for SBRT for lung cancer. This study shows that PredDose can be used to automatically create treatment plans for SBRT in early‐stage lung cancer. Various methods have been developed for automated VMAT treatment planning using machine learning. In the case of commercial software, treatment planning is created by performing optimization calculations based on the DVH predicted based on the CT image and its contours.^37^ Other methods can also predict the start and stop angles of the gantry angle and the parameters of the objective function from the CT and contour and use them to create a treatment plan.^19^ In a previous study, CT images and contours were used as inputs for the DL model to predict the MLC aperture and MU for all control points; the RT plan was then created based on these predictions.^38^ In this study, we used ring‐shaped dose structures converted from the PredDose generated by the DL model and compared them to these techniques. This is the first study to demonstrate that it is possible to automatically create RT plans for early‐stage lung cancer that reproduce the dose distribution predicted using this optimization method. Optimization using ring‐shaped dose structures was performed using only the vendor‐supplied objective functions, as listed in Table 2. Therefore, this optimization method using RatoGuide has the potential to be applied in various treatment planning systems. Furthermore, if the outcomes produced by automated planning are unsatisfactory, it is feasible to perform manual fine‐tuning.

All automated treatment plans were evaluated as acceptable for each case. However, it is unclear whether they can be approved by radiation oncologists at other facilities or applied to treatment plans with other prescribed doses. The predicted dose can be normalized to meet the prescribed dose for the target structure using the RatoGuide functions. However, if unacceptable dose constraints for OARs remain, it is necessary to develop and evaluate models that are trained on the treatment plans of each facility and different dose prescriptions and models that output multiple dose distributions for each prescription dose or type. The judgment on qualitative evaluation was divided among the radiation oncologists. Given actual clinical practice after AI dissemination in the future, planners who can check dose distribution maps in more detail and make minor modifications after checking will be ideal.

Our study had several limitations. First, all the treatment plans in this study were generated using a single coplanar partial arc. This beam arrangement was selected to reduce the beam‐on time during inspiration breath‐holding and limit the radiation dose to the contralateral lung. According to the JCOG1408 and JROSG10‐1 protocols, the use of coplanar beams is acceptable. Because all the clinical VMAT plans used to treat patients in this study were created using only coplanar beams, the same beam arrangement was used for the automated plans. Our findings revealed that 100% of automated plans met the RTOG CI criteria^25^, ^26^ For RTOG R50 criteria^25^, ^26^ the compliance rate was 100% for peripheral cases and 88% for central cases when minor deviations were included. However, the potential dosimetric benefits of alternative beam arrangements, including full arcs, multiple arcs, and noncoplanar beams, were not examined. A previous study has shown that noncoplanar techniques can improve dose conformity such as R50 and CI values^39^ Further research is required to determine whether these alternative configurations could improve the quality of treatment plans. In the future, creating training datasets based on treatment plans that include other beam arrangements may allow the development of AI models that meet the more stringent dose conformity requirements described in protocols such as RTOG 0236, 0813, and 0915.^24^, ^25^, ^26^, ^40^ Second, the numbers of training and test cases were small. However, the results of this study demonstrated the possibility of creating deliverable VMAT plans that fully satisfy the dose constraints defined by JCOG1408 and JROSG10‐1. Third, further studies are required to determine whether this model can be applied to other facilities. Fourth, the parameters of the ring‐shaped dose structures were set according to vendor recommendations. The impact of these settings on the plan quality is uncertain and should be evaluated in future studies. Further studies are required to improve the optimization method and reproduce PredDose. Fifth, we created and evaluated the dose distribution prediction models limited to SBRT for early‐stage lung cancer to predict the dose distribution with high accuracy. Further studies are required to evaluate the quality of other types of treatment planning and external datasets. Finally, further studies are required to assess the accuracy of beam delivery using the automated treatment plan developed in this study.

## 5 CONCLUSION

These results indicate the feasibility of automated treatment planning using DL algorithms and suggest that DL can be used to create clinically acceptable SBRT‐VMAT treatment plans for lung cancer efficiently. We will continue to investigate methods to improve the accuracy of the prediction model and overall workflow.

## AUTHOR CONTRIBUTIONS

**Hikaru Nemoto**: Conceptualization; data curation; formal analysis; methodology; project administration; resources; software; writing—original draft preparation; writing—review and editing. **Masahide Saito**: Formal analysis; methodology; project administration; software; writing—original draft preparation; writing—review and editing. **Noriyuki Kadoya**: Conceptualization; formal analysis; methodology; project administration; supervision; writing—original draft preparation. **Takafumi Komiyama**: Data curation. **Ryota Tozuka**: Data curation; software. **Hiroshi Onishi**: Methodology; project administration; resources; writing—review and editing. **Keiichi Jingu**: Conceptualization; methodology; project administration; resources; supervision; writing—original draft preparation; writing—review and editing.

## CONFLICT OF INTEREST STATEMENT

Noriyuki Kadoya and Ryota Tozuka owned stock in AiRato Inc., while Masahide Saito, Hiroshi Onishi, and Keiichi Jingu received grants from AiRato Inc.

## ETHICS STATEMENT

This retrospective study was approved by the institutional review board (IRB) of the University of Yamanashi (receipt number: CS0010).

## Supporting information



Supporting Information

## References

[1] Tol JP , Doornaert P , Witte BI , Dahele M , Slotman BJ , Verbakel WF . A longitudinal evaluation of improvements in radiotherapy treatment plan quality for head and neck cancer patients. Radiother Oncol. 2016;119(2):337‐343. doi:10.1016/j.radonc.2016.04.011 27130730 10.1016/j.radonc.2016.04.011

[2] Verbakel WF , Cuijpers JP , Hoffmans D , Bieker M , Slotman BJ , Senan S . Volumetric intensity‐modulated arc therapy vs. conventional IMRT in head‐and‐neck cancer: a comparative planning and dosimetric study. Int J Radiat Oncol Biol Phys. 2009;74(1):252‐259. doi:10.1016/j.ijrobp.2008.12.033 19362244 10.1016/j.ijrobp.2008.12.033

[3] Jiang X , Li T , Liu Y , et al. Planning analysis for locally advanced lung cancer: dosimetric and efficiency comparisons between intensity‐modulated radiotherapy (IMRT), single‐arc/partial‐arc volumetric modulated arc therapy (SA/PA‐VMAT). Radiat Oncol. 2011;6:1‐7. doi:10.1186/1748‐717X‐6‐140 22014217 10.1186/1748-717X-6-140 PMC3207896

[4] Holt A , van Vliet‐Vroegindeweij C , Mans A , Belderbos JS , Damen EM . Volumetric‐modulated arc therapy for stereotactic body radiotherapy of lung tumors: a comparison with intensity‐modulated radiotherapy techniques. Int J Radiat Oncol Biol Phys. 2011;81(5):1560‐1567. doi:10.1016/j.ijrobp.2010.09.014 21300461 10.1016/j.ijrobp.2010.09.014

[5] Ong CL , Verbakel WF , Cuijpers JP , Slotman BJ , Lagerwaard FJ , Senan S . Stereotactic radiotherapy for peripheral lung tumors: a comparison of volumetric modulated arc therapy with 3 other delivery techniques. Radiother Oncol. 2010;97(3):437‐442. doi:10.1016/j.radonc.2010.09.027 21074878 10.1016/j.radonc.2010.09.027

[6] Okamoto H , Wakita A , Tani K , et al. Plan complexity metrics for head and neck VMAT competition plans. Med Dosim. 2024;49(3):244‐253. doi:10.1016/j.meddos.2024.01.007 38368182 10.1016/j.meddos.2024.01.007

[7] Okamoto H , Murakami N , Isohashi F , et al. Plan quality association between dummy run and individual case review in a prospective multi‐institutional clinical trial of postoperative cervical cancer patients treated with intensity‐modulated radiotherapy: Japan clinical Oncology Group study (JCOG1402). Radiother Oncol. 2023;183:109630. 36934892 10.1016/j.radonc.2023.109630

[8] Das IJ , Cheng CW , Chopra KL , Mitra RK , Srivastava SP , Glatstein E . Intensity‐modulated radiation therapy dose prescription, recording, and delivery: patterns of variability among institutions and treatment planning systems. J Natl Cancer Inst. 2008;100(5):300‐307. doi:10.1093/jnci/djn020 18314476 10.1093/jnci/djn020

[9] Nelms BE , Robinson G , Markham J , et al. Variation in external beam treatment plan quality: an inter‐institutional study of planners and planning systems. Pract Radiat Oncol. 2012;2(4):296‐305. doi:10.1016/j.prro.2011.11.012 24674168 10.1016/j.prro.2011.11.012

[10] Kajikawa T , Kadoya N , Ito K , et al. A convolutional neural network approach for IMRT dose distribution prediction in prostate cancer patients. J Radiat Res. 2019;60(5):685‐693. doi:10.1093/jrr/rrz051 31322704 10.1093/jrr/rrz051 PMC6805973

[11] Duan Y , Wang J , Wu P , et al. AS‐NeSt: a novel 3D deep learning model for radiation therapy dose distribution prediction in esophageal cancer treatment with multiple prescriptions. Int J Radiat Oncol Biol Phys. 2024;119(3):978‐989. doi:10.1016/j.ijrobp.2023.12.001 38159780 10.1016/j.ijrobp.2023.12.001

[12] Koike Y , Takegawa H , Anetai Y , Ohira S , Nakamura S , Tanigawa N . Patient‐specific three‐dimensional dose distribution prediction via deep learning for prostate cancer therapy: improvement with the structure loss. Phys Med. 2023;107:102544. doi:10.1016/j.ejmp.2023.102544 36774846 10.1016/j.ejmp.2023.102544

[13] Shen Y , Tang X , Lin S , Jin X , Ding J , Shao M . Automatic dose prediction using deep learning and plan optimization with finite‐element control for intensity modulated radiation therapy. Med Phys. 2024;51(1):545‐555. doi:10.1002/mp.16743 37748133 10.1002/mp.16743

[14] Church C , Yap M , Bessrour M , Lamey M , Granville D . Automated plan generation for prostate radiotherapy patients using deep learning and scripted optimization. Phys Imaging Radiat Oncol. 2024;32:100641. doi:10.1016/j.phro.2024.100641 39310221 PMC11415801

[15] Wang N , Fan J , Xu Y , et al. Clinical implementation and evaluation of deep learning‐assisted automatic radiotherapy treatment planning for lung cancer. Phys Med. 2024;124:104492. doi:10.1016/j.ejmp.2024.104492 39094213 10.1016/j.ejmp.2024.104492

[16] Wang H , Bai X , Wang Y , Lu Y , Wang B . An integrated solution of deep reinforcement learning for automatic IMRT treatment planning in non‐small‐cell lung cancer. Front Oncol. 2023;13:1124458. doi:10.3389/fonc.2023.1124458 36816929 10.3389/fonc.2023.1124458 PMC9936236

[17] Kadoya N , Kimura Y , Tozuka R , et al. Evaluation of deep learning‐based deliverable VMAT plan generated by prototype software for automated planning for prostate cancer patients. J Radiat Res. 2023;64(5):842‐849. doi:10.1093/jrr/rrad058 37607667 10.1093/jrr/rrad058 PMC10516733

[18] Saito M , Kadoya N , Kimura Y , et al. Evaluation of deep learning based dose prediction in head and neck cancer patients using two different types of input contours. J Appl Clin Med Phys. 2024;25:e14519. doi:10.1002/acm2.14519 39285649 10.1002/acm2.14519 PMC11633794

[19] Bai X , Shan G , Chen M , Wang B . Approach and assessment of automated stereotactic radiotherapy planning for early stage non‐small‐cell lung cancer. Biomed Eng Online. 2019;18(1):101. doi:10.1186/s12938‐019‐0721‐7 31619263 10.1186/s12938-019-0721-7 PMC6796412

[20] Kimura T , Nagata Y , Eba J , et al. A randomized Phase III trial of comparing two dose‐fractionations stereotactic body radiotherapy (SBRT) for medically inoperable Stage IA non‐small cell lung cancer or small lung lesions clinically diagnosed as primary lung cancer: Japan Clinical Oncology Group Study JCOG1408 (J‐SBRT trial). Jpn J Clin Oncol. 2017;47(3):277‐281. 28073946 10.1093/jjco/hyw198

[21] Kimura T , Nagata Y , Harada H , et al. Phase I study of stereotactic body radiation therapy for centrally located stage IA non‐small cell lung cancer (JROSG10‐1). Int J Clin Oncol. 2017;22(5):849‐856. doi:10.1007/s10147‐017‐1125‐y 28466183 10.1007/s10147-017-1125-y

[22] Nguyen D , Jia X , Sher D , et al. 3D radiotherapy dose prediction on head and neck cancer patients with a hierarchically densely connected U‐net deep learning architecture. Phys Med Biol. 2019;64(6):065020. doi:10.1088/1361‐6560/ab039b 30703760 10.1088/1361-6560/ab039b

[23] Paddick I . A simple scoring ratio to index the conformity of radiosurgical treatment plans. J Neurosurg. 2000;93(suppl 3):219‐222. doi:10.3171/jns.2000.93.supplement_3.0219 11143252 10.3171/jns.2000.93.supplement

[24] Timmerman R , Paulus R , Galvin J , et al. Stereotactic body radiation therapy for inoperable early stage lung cancer. JAMA. 2010;303(11):1070‐1076. doi:10.1001/jama.2010.261 20233825 10.1001/jama.2010.261 PMC2907644

[25] Bezjak A , Paulus R , Gaspar LE , et al. Safety and efficacy of a five‐fraction stereotactic body radiotherapy schedule for centrally located non‐small‐cell lung cancer: NRG oncology/RTOG 0813 trial. J Clin Oncol. 2019;37(15):1316‐1325. doi:10.1200/JCO.18.00622 30943123 10.1200/JCO.18.00622 PMC6524984

[26] Videtic GM , Hu C , Singh AK , et al. A randomized phase 2 study comparing 2 stereotactic body radiation therapy schedules for medically inoperable patients with stage I peripheral non‐small cell lung cancer: NRG oncology RTOG 0915 (NCCTG N0927). Int J Radiat Oncol Biol Phys. 2015;93(4):757‐764. doi:10.1016/j.ijrobp.2015.07.2260 26530743 10.1016/j.ijrobp.2015.07.2260 PMC4744654

[27] Ventura T , Dias J , Khouri L , et al. Clinical validation of a graphical method for radiation therapy plan quality assessment. Radiat Oncol. 2020;15:1‐10. doi:10.1186/s13014‐020‐01507‐5 10.1186/s13014-020-01507-5 PMC7068922 32164752

[28] Glatzer M , Panje CM , Sirén C , Cihoric N , Putora PM . Decision making criteria in oncology. Oncology. 2020;98(6):370‐378. doi:10.1159/000492272 30227426 10.1159/000492272

[29] Sun Z , Xia X , Fan J , et al. A hybrid optimization strategy for deliverable intensity‐modulated radiotherapy plan generation using deep learning‐based dose prediction. Med Phys. 2022;49(3):1344‐1356. doi:10.1002/mp.15462 35043971 10.1002/mp.15462

[30] Brodin NP , Schulte L , Velten C , et al. Organ‐at‐risk dose prediction using a machine learning algorithm: clinical validation and treatment planning benefit for lung SBRT. J Appl Clin Med Phys. 2022;23(6):e13609. doi:10.1002/acm2.13609 35460150 10.1002/acm2.13609 PMC9195027

[31] Visak J , McGarry RC , Randall ME , Pokhrel D . Development and clinical validation of a robust knowledge‐based planning model for stereotactic body radiotherapy treatment of centrally located lung tumors. J Appl Clin Med Phys. 2021;22(1):146‐155. doi:10.1002/acm2.13120 10.1002/acm2.13120 PMC7856508 33285034

[32] Visak J , Ge GY , McGarry RC , Randall M , Pokhrel D . An Automated knowledge‐based planning routine for stereotactic body radiotherapy of peripheral lung tumors via DCA‐based volumetric modulated arc therapy. J Appl Clin Med Phys. 2021;22(1):109‐116. doi:10.1002/acm2.13114 33270975 10.1002/acm2.13114 PMC7856484

[33] Hof SV , Delaney AR , Tekatli H , et al. Knowledge‐based planning for identifying high‐risk stereotactic ablative radiation therapy treatment plans for lung tumors larger than 5 cm. Int J Radiat Oncol Biol Phys. 2019;103(1):259‐267. doi:10.1016/j.ijrobp.2018.08.013 30114461 10.1016/j.ijrobp.2018.08.013

[34] Wortel G , Eekhout D , Lamers E , et al. Characterization of automatic treatment planning approaches in radiotherapy. Phys Imaging Radiat Oncol. 2021;19:60‐65. doi:10.1016/j.phro.2021.07.003 34307920 10.1016/j.phro.2021.07.003 PMC8295841

[35] Kubo K , Monzen H , Ishii K , et al. Dosimetric comparison of RapidPlan and manually optimized plans in volumetric modulated arc therapy for prostate cancer. Physica Medica. 2017;44:199‐204. doi:10.1016/j.ejmp.2017.06.026 28705507 10.1016/j.ejmp.2017.06.026

[36] Mitchell RA , Wai P , Colgan R , Kirby AM , Donovan EM . Improving the efficiency of breast radiotherapy treatment planning using a semi‐automated approach. J Appl Clin Med Phys. 2017;18(1):18‐24. doi:10.1002/acm2.12006 28291912 10.1002/acm2.12006 PMC5689888

[37] Li N , Carmona R , Sirak I , et al. Highly efficient training, refinement, and validation of a knowledge‐based planning quality‐control system for radiation therapy clinical trials. Int J Radiat Oncol Biol Phys. 2017;97(1):164‐172. doi:10.1016/j.ijrobp.2016.10.005 27979445 10.1016/j.ijrobp.2016.10.005 PMC5175211

[38] Vandewinckele L , Reynders T , Weltens C , Maes F , Crijns W . Deep learning based MLC aperture and monitor unit prediction as a warm start for breast VMAT optimisation. Phys Med Biol. 2023;68(22):225013. doi:10.1088/1361‐6560/ad07f6 10.1088/1361-6560/ad07f6 37903442

[39] Kei T , Luca K , Kayode O , et al. Improving lung Stereotactic Body Radiation Therapy dose conformity using a simple noncoplanar Volumetric Modulated Arc Therapy technique. Pract Radiat Oncol. 2025;15(4):e332‐e338. doi:10.1016/j.prro.2025.02.007 40015603 10.1016/j.prro.2025.02.007

[40] Timmerman R , Galvin J , Michalski J , et al. Accreditation and quality assurance for Radiation Therapy Oncology Group: multicenter clinical trials using Stereotactic Body Radiation Therapy in lung cancer. Acta oncologica. 2006;45(7):779‐786. doi:10.1080/02841860600902213 16982540 10.1080/02841860600902213

