# Supplementary material for: Evaluation of deep learning‐based automated radiotherapy planning for early‐stage lung cancer using SBRT‐VMAT: A comparison with manual planning
Source: J Appl Clin Med Phys. 2025 Oct 14;26(10):e70291. doi: 10.1002/acm2.70291 (PMC12521067; doi:10.1002/acm2.70291)
Supplement: Supplementary file 1 — Supporting Information [file ACM2-26-e70291-s001.docx]

**Supplemental document**

Table S1. Function values displayed in RayStation at the end of optimization for each objective used to generate the script-based automated plan. This table shows the normalized function values based on the total function value for each case, along with the average values for each structure. The top three highest values are highlighted in red.

1. Peripheral case

| **Structure** | **Objectives** | **Description** | **Function value at the end of optimization (Normalized)** | | | | | | | | |
| --- | --- | --- | --- | --- | --- | --- | --- | --- | --- | --- | --- |
|  |  |  | **Test case1** | **Test case2** | **Test case3** | **Test case4** | **Test case5** | **Test case6** | **Test case7** | **Test case8** | **Mean** |
| PTV | Min DVH | 5500cGy to 95 % volume | 2.01E-13 | 0.00E+00 | 0.00E+00 | 0.00E+00 | 2.18E-08 | 2.79E-09 | 0.00E+00 | 0.00E+00 | 3.07E-09 |
| PTV | Max dose | 6875cGy | 5.81E-01 | 3.69E-01 | 6.20E-01 | 6.41E-01 | 5.66E-01 | 4.82E-01 | 5.27E-01 | 7.32E-01 | 5.65E-01 |
| Dose structure_100.0_120.0% | Min DVH | 6050cGy to 50 % volume | 0.00E+00 | 0.00E+00 | 1.62E-04 | 0.00E+00 | 0.00E+00 | 0.00E+00 | 0.00E+00 | 0.00E+00 | 2.03E-05 |
| Dose structure_80.0_100.0% | Max dose | 5500cGy | 8.98E-02 | 4.92E-02 | 1.55E-02 | 1.26E-01 | 3.65E-02 | 3.03E-02 | 1.22E-02 | 3.86E-02 | 4.97E-02 |
| Dose structure_60.0_80.0% | Max dose | 4400cGy | 7.40E-02 | 2.37E-02 | 2.06E-02 | 7.98E-02 | 1.29E-02 | 2.44E-02 | 7.53E-02 | 6.24E-02 | 4.66E-02 |
| Dose structure_40.0_60.0% | Max dose | 3300cGy | 7.69E-02 | 5.84E-02 | 3.93E-02 | 6.76E-02 | 1.73E-02 | 8.82E-02 | 8.10E-02 | 5.56E-02 | 6.05E-02 |
| Dose structure_20.0_40.0% | Max dose | 2200cGy | 8.82E-02 | 1.95E-01 | 1.08E-01 | 4.02E-02 | 8.50E-02 | 1.72E-01 | 1.22E-01 | 3.58E-02 | 1.06E-01 |
| Dose structure_0.0_20.0% | Max dose | 1100cGy | 8.99E-02 | 3.04E-01 | 1.96E-01 | 4.56E-02 | 2.83E-01 | 2.04E-01 | 1.83E-01 | 7.53E-02 | 1.73E-01 |

1. Central case

| **Structure** | **Objectives** | **Description** | **Function value at the end of optimization (Normalized)** | | | | | | | | |
| --- | --- | --- | --- | --- | --- | --- | --- | --- | --- | --- | --- |
|  |  |  | **Test case1** | **Test case2** | **Test case3** | **Test case4** | **Test case5** | **Test case6** | **Test case7** | **Test case8** | **Mean** |
| PTV | Min DVH | 5000cGy to 95 % volume | 0.00E+00 | 0.00E+00 | 0.00E+00 | 6.43E-11 | 2.49E-08 | 0.00E+00 | 2.42E-10 | 2.26E-13 | 3.15E-09 |
| PTV | Max dose | 6000cGy | 0.00E+00 | 1.57E-02 | 4.87E-01 | 0.00E+00 | 0.00E+00 | 3.25E-01 | 7.24E-01 | 5.30E-01 | 2.60E-01 |
| Dose structure_100.0_120.0% | Min DVH | 5500cGy to 50 % volume | 1.74E-03 | 0.00E+00 | 0.00E+00 | 0.00E+00 | 2.02E-03 | 0.00E+00 | 2.57E-04 | 3.07E-05 | 5.05E-04 |
| Dose structure_80.0_100.0% | Max dose | 5000cGy | 6.58E-02 | 6.86E-02 | 3.60E-02 | 2.94E-01 | 1.14E-01 | 1.69E-01 | 4.17E-02 | 1.84E-02 | 1.01E-01 |
| Dose structure_60.0_80.0% | Max dose | 4000cGy | 1.58E-01 | 1.77E-01 | 6.52E-02 | 3.10E-01 | 1.38E-01 | 1.40E-01 | 7.80E-02 | 1.40E-01 | 1.51E-01 |
| Dose structure_40.0_60.0% | Max dose | 3000cGy | 2.80E-01 | 3.25E-01 | 1.23E-01 | 2.26E-01 | 1.82E-01 | 1.31E-01 | 4.60E-02 | 8.93E-02 | 1.75E-01 |
| Dose structure_20.0_40.0% | Max dose | 2000cGy | 2.06E-01 | 1.91E-01 | 1.45E-01 | 3.31E-02 | 2.92E-01 | 1.31E-01 | 4.39E-02 | 7.34E-02 | 1.39E-01 |
| Dose structure_0.0_20.0% | Max dose | 1000cGy | 2.89E-01 | 2.22E-01 | 1.45E-01 | 1.37E-01 | 2.72E-01 | 1.04E-01 | 6.59E-02 | 1.49E-01 | 1.73E-01 |
